# In-Lake Processes Offset Increased Terrestrial Inputs of Dissolved Organic Carbon and Color to Lakes

**DOI:** 10.1371/journal.pone.0070598

**Published:** 2013-08-15

**Authors:** Stephan J. Köhler, Dolly Kothawala, Martyn N. Futter, Olof Liungman, Lars Tranvik

**Affiliations:** 1 SLU Department of Aquatic Sciences and Assessment, Uppsala, Sweden; 2 Evolutionary Biology Center, Department of Limnology, Uppsala, Uppsala, Sweden; 3 DHI Water and Environment Pty Ltd. St George's Terrace, Sydney, Australia; Utrecht University, Netherlands

## Abstract

Increased color in surface waters, or browning, can alter lake ecological function, lake thermal stratification and pose difficulties for drinking water treatment. Mechanisms suggested to cause browning include increased dissolved organic carbon (DOC) and iron concentrations, as well as a shift to more colored DOC. While browning of surface waters is widespread and well documented, little is known about why some lakes resist it. Here, we present a comprehensive study of Mälaren, the third largest lake in Sweden. In Mälaren, the vast majority of water and DOC enters a western lake basin, and after approximately 2.8 years, drains from an eastern basin. Despite 40 years of increased terrestrial inputs of colored substances to western lake basins, the eastern basin has resisted browning over this time period. Here we find the half-life of iron was far shorter (0.6 years) than colored organic matter (A_420_ ; 1.7 years) and DOC as a whole (6.1 years). We found changes in filtered iron concentrations relate strongly to the observed loss of color in the western basins. In addition, we observed a substantial shift from colored DOC of terrestrial origin, to less colored autochthonous sources, with a substantial decrease in aromaticity (-17%) across the lake. We suggest that rapid losses of iron and colored DOC caused the limited browning observed in eastern lake basins. Across a wider dataset of 69 Swedish lakes, we observed greatest browning in acidic lakes with shorter retention times (< 1.5 years). These findings suggest that water residence time, along with iron, pH and colored DOC may be of central importance when modeling and projecting changes in brownification on broader spatial scales.

## Introduction

Browning of surface waters has been reported from many studies across the northern hemisphere [1] It has been ascribed to increases in dissolved organic carbon (DOC) concentrations [2], increases in total iron [3], and changes in the light absorbing properties of dissolved organic matter (DOM) [4]. Multiple and possibly not mutually exclusive reasons for increased transport of DOC to surface waters have been presented, including drier climate [5], wetter climate [6], warmer temperatures [7] or declines in acid deposition [2]. Iron and its interactions with DOC are known to increase the color of surface waters [8,9,10,11]. With increasing pH, iron will be bound strongly to colloidal organic matter [12], occur as colloidal ferrihydrate [13] or both [14]. These transformations of iron speciation may have profound consequences on both the concentration of DOC and color of surface waters. Increasing water color is a serious concern in regions that are dependent on surface waters for drinking water supply [15] as the cost of water treatment may increase, and chlorination of organic matter may produce carcinogenic compounds [16]. Darker water will alter lake thermal properties [17,18] and potentially increase the number of algal blooms [19] or profoundly affect lake productivity at several trophic levels [20]. Inland waters have been recognized as an important source of carbon in the global carbon cycle [21] increasing DOC concentrations may change the internal carbon turnover in lakes and increase inorganic carbon fluxes to the atmosphere [22]. Removal of DOC from lake water may be due to microbial mineralization [23], flocculation [24] or photolytic processing [25,26]. The magnitude of these in-lake processes is constrained by water residence time (WRT) [27,28,29,9].

**Figure 1:**
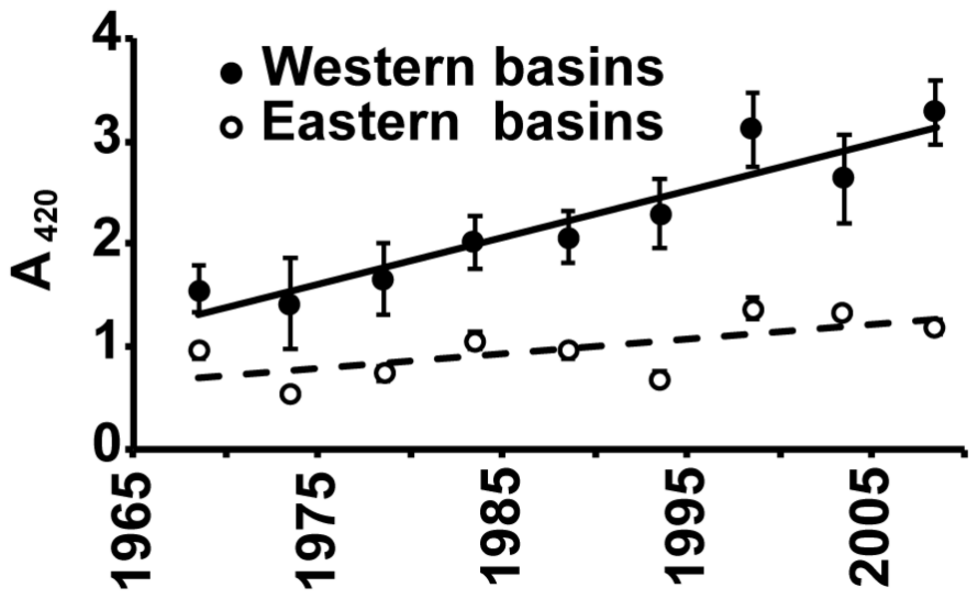
Change in absorbance measured at 420nm (A_420_) in the three sampling points within the Western Basins A and B (•), and three sampling sites within the Eastern Basins C and E (ο) calculated as five year averages over 45 years (between 1967 and 2011).

Here, we attempt to disentangle the processes that control changes in color, DOC and iron in lakes using a long-term data set from Sweden’s third largest lake (Mälaren). Historical records show that water color in the major eastern basin of Mälaren, Sweden’s third largest lake, has increased less than 25% over the last 40 years (Figure 1). This is surprising since the corresponding changes in color in the western basins, where the majority of water enters the lake (> 70%), were higher than 100% in the same time period. Mälaren consists of several connected basins that form a natural gradient of increasing WRT with movement from west to east. On average, water in the lake outlet has been resident within the lake for 2.8 years. This natural gradient in WRT and the availability of long-term monitoring data makes Mälaren an ideal site for studying processes and rates of change to lake color and DOC. Here, we examine the extent and factors contributing to the change of DOC and color across the basins of Mälaren, and compare the results with a set of 69 intensively studied lakes from across Sweden with varying WRT.

## Material and Methods

### Ethics Statement

Lake and river water samples were collected at multiple sites across Mälaren as part of a routine monitoring program ordered by the joint water authorities around Mälaren [30]. All sites have public access and are also part of the *Swedishmonitoring* program. Thus no specific permissions were required for these and the above locations. Our field studies did not involve endangered or protected species.

### Study Sites and Sampling

**Figure 2:**
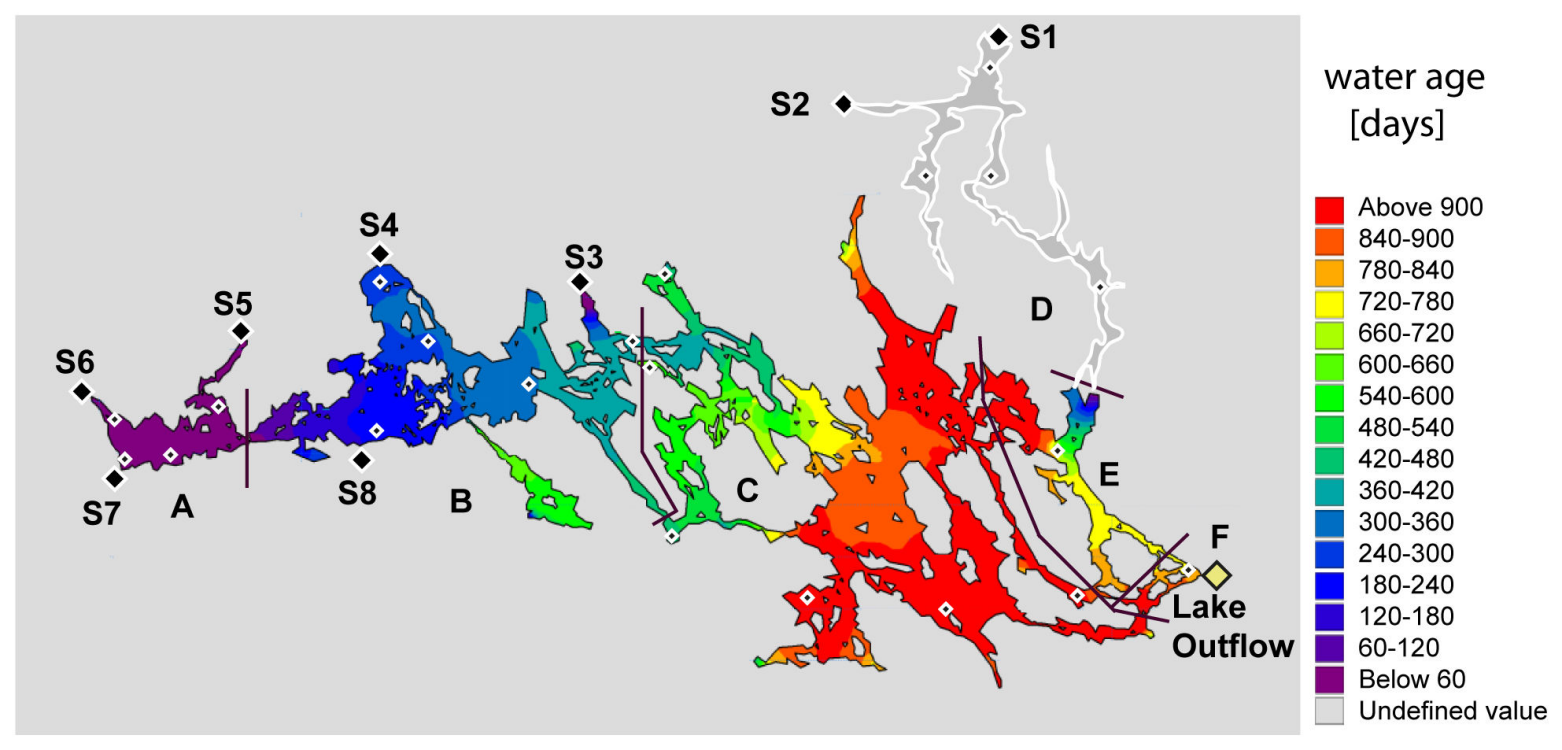
Spatial variability of the modeled water residence time of Mälaren, with the age of water increasing from western to eastern basins. Lake basins are identified by letters (A–F), with lake sites (white diamonds) and streams sites (black diamonds, S1-S8). Basin D is identified in grey as it was not included in the hydrological water residence time model.

Mälaren (surface area 1120 km^2^, 59°30’ N 17° 12’ E) supplies drinking water to approximately 1.5 million residents in Stockholm and surrounding communities. Mälaren is subdivided into six major basins (A-F in Figure 2), and has 12 river inputs. The mean depth is 12.8 m, with the deepest basins (>40 m) in the east (basin C, Figure 2).

Water samples were collected from 23 lake and 8 river sites (Figure 2). Epilimnetic lake water samples were collected at a depth of 1m by lowering bottles from a helicopter, while river inlets were sampled by hand from mid-stream where possible, otherwise from shore. Sampling occurred on Aug 23 2010. During August, all lake basins were thermally stratified, water temperatures were near annual maximum values, and incident solar radiation was past June/July maxima. All samples were collected in pre-washed 1 or 2L polyethylene bottles and stored in the dark at 4° C. While this study focuses on results from water sampling in August 2010, we draw upon supporting chemical and hydrological data compiled from sites within long-term sampling campaigns as part of the Swedish surface water monitoring program [31]. The long-term Mälaren survey includes 11 of the 23 lake sites included in the August 2010 sampling, and six stream sites. These six streams collectively contribute ~70% of the annual water budget [32] to Mälaren, and drain into western lake basins A and B (Figure 2). Inputs from the remainder of the Mälaren catchment were not gauged but were pro-rated to the gauged inputs assuming similar per area carbon export as the six gauged streams (see SI for details). The main outflow is at the south-eastern end of the lake (Figure 2).

### Hydrological model for water residence times

A 3D hydrodynamic model (see SI for details) has been applied to Mälaren in order to investigate the circulation and exchange between different sub-basins [33]. The model was run for the year 2007 [33], using observed ice cover as input during the winter months. The model was calibrated and validated against observed temperature profiles at a number of stations, observed water levels at two stations and the observed combined outflows through Stockholm and using an internal age tracer. Simulated age of surface waters in the different basins is shown in Figure 2. The lower values at the northeastern end of Mälaren are due to an inflow (sampling basin D in Figure 2), and this water is considered juvenile (age zero).

### Laboratory Analysis

Water chemistry was analyzed as part of a routine Swedish monitoring program at the Swedish University of Agricultural Sciences. All August 2010 lake and stream samples as well as routine monitoring samples were analyzed for pH, absorbance (A_420_) total organic carbon (TOC), total nitrogen (TN), nitrate (NO_3_^-^) and ammonium (NH_4_^+^) using accredited methods [31]. Organic nitrogen concentrations were calculated as the difference between TN and the sum of NO_3_^-^ and NH_4_^+^. In addition to the above routine analysis, August 2010 samples were filtered with glass fiber filters (Whatman GF/C, effective pore size, ≈1.2 µm) within a few hours of collection, and all chemical and optical analyses were performed within a week of collection. Filtered and unfiltered samples were analyzed using a Shimadzu TOC-5000 analyzer by the NPOC (non-purgable organic carbon) method. Unfiltered NPOC concentrations represent total organic carbon (TOC) and filtered concentrations represent DOC. The Swedish national monitoring program has been reporting absorbance at a single wavelength of 420 nm (A_420_) since 1967 using filtered water samples.

### Analysis of dissolved iron

Iron (Fe) was analyzed on filtered water samples. Iron passing the filter can exist as truly dissolved monomeric inorganic iron complexes or as one of two colloidal forms, i) as ferrihydrate and ii) iron that is bound to organic matter [12,12,34]. Monomeric inorganic iron complexes may be quantified by measurements made before and after passage through a cation exchange column [26] as inorganically bound iron will remain in the column. Using this procedure, no inorganically bound iron was detected in any of our samples (See File S1 for details on the exchange column experiment and Figure S1). Both colloidal forms are associated with organic matter. Herein, we will refer to the sum of those two forms as colloidal iron (Fe_coll_).

### Fluorescence

Filtered water samples were analyzed within three days of collection in a 1 cm quartz cuvette using a fluorescence spectrophotometer (SPEX FluoroMax-2, Horiba Jobin Yvon) to acquire excitation-emission matrices (EEM). Excitation wavelengths (λ_ex_) spanned from 250–445 nm, at 5 nm increments, emission wavelengths (λ_em_) spanned from 300–600 nm, at 4 nm increments, slit widths were set to 5 nm, with an integration time of 0.1 seconds. We calculated the fluorescence index (FI) [35,36] using the ratio of intensities at λ_em_ 470 and 520 nm, at an λ_ex_ of 370 nm, and the freshness index (β: α) [37], as the ratio of intensities at λ_em_ 380 nm divided by the λ_em_ maximum between 420 and 435, at an λ_ex_ of 310 nm. The humification index (HIX) was measured as the ratio of the areas under the emission curve between 435 to 480 and 300 to 445 nm at an excitation of 254 nm [38]. The location (λ_ex:_ λ_em_) and fluorescence intensities of four peaks, Peaks A (250:448), C (330:432), T (300:400) and M (270:308) were identified [39].

### Absorbance

Absorbance spectra of filtered lake water samples were measured using a Lambda 40 UV-visible spectrophotometer (PerkinElmer, Waltham, USA) in a 1 cm or 5 cm quartz cuvette spanning from λ’ s 200-800 nm at 1 nm intervals, slit width of 2 nm, and scan speed of 240 nm min^-1^, and all spectra were corrected for potential nitrate interferences (see 40). All absorbance intensities at a particular wavelength (A_λ_) are expressed here as m^-1^. The specific absorbance (SUVA in L mg C^-1^ m^-1^) was calculated by normalizing A_λ_ to DOC concentration, and % aromaticity was calculated from SUVA using a relationship from [11].

### High Performance Size Exclusion Chromatography (HPSEC)

HPSEC was used to determine the molecular weight (MW) distribution of DOC, using a Gilson 321 pump and controller with a flow rate of 0.5 ml min^-1^, and a 155 UV-absorbance detector set to 254 nm (Gilson Inc., Middleton, WI), using an injection volume of 100 µL according to [41] (see SI for a detailed description). The apparent MW is estimated from peak retention time and based on polystyrene sulfonate MW standards. We divided apparent MW into very large (> 4 kDa), large (1-4 kDa), medium (0.2-1 kDa) and small (<0.2 Da) size fractions.

### Estimating rates of change and half-life of DOC

To estimate loss rates for DOC and other spectral characteristics (A_420_, A_254_, Fe_coll_, SUVA_420_, SUVA_254_, DOC_FI_auto_, DOC_FI_mport,_ FI, Freshness Index, and MW fractions) over time we used the function ln(predicted) = *b*_*0*_ + k * age, and converted this relationship to the exponential function (predicted= e^b0^ * e^k* age^), where *k* is the exponential rate of change over time with units yr^-1^, and *b*_*0*_ is a fitting parameter. The resulting relationships were used to calculate the amount of each variable at WRT of 0 and 2.8 years, the average age of water at the Mälaren outlet (*t*_*0*_ and *t*_*2.8*_). The half life (*t*_*1/2*_) was calculated as ln (2) /*k*.

### Estimates of color sources within the Mälaren basin

The observed changes in chemical and spectroscopic properties across Mälaren may be due, in part, to internal processing of terrestrially-derived DOC and autochthonous (in-lake) production of DOC. Here we use two approaches to interpolate the proportion of DOC entering from the surrounding catchment via streams (DOC_import_) and autochthonous production of new DOC (DOC_auto_). The first approach is based on the Fluorescence Index (FI), where a FI of 1.2 is representative of terrestrial DOC (DOC_FI_import_), and 1.8 represents DOC produced within the lake (DOC_FI_auto_) [35], and assuming that FI changes linearly according to the contributions from two end members. We follow [35] in noting that the FI is not specific enough to differentiate between sources when the change in FI is small (< 0.1).

The second absorbance model is based on an empirical fit of measured variables to modeled absorbance (A_λ_) of lake water, at 254 or 420 nm (A_254_, A_420_). Again we assume that the measured lake DOC (DOC_Sum_) was comprised of two sources, external stream derived inputs that are primarily of terrestrial origin (DOC_import_), and autochthonous internally derived DOC (DOC_auto_) (eq. 1).

$$DOC_{Sum}=DOC_{Import}+DOC_{auto}$$ (1)

The DOC_auto_ pool was expressed as a function of the freshness index (β: α) and total DOC (eq. 2).

$$DOC_{auto}=d\times \left(\frac{\beta }{\alpha }\right)\times DOC_{Sum}$$ (2)

We note that the freshness index was strongly related to WRT (*R* ^2^ =0.90, *p* < 0.0001). The A_λ_ was predicted from the proportion of two DOC pools, DOC_import_ and DOC_auto_, along with iron associated to colloidal organic matter (Fe_coll_) (Eq. 3).

$$A_{\lambda }^{Modelled}=a\times DOC_{import}+b\times Fe_{coll}+c\times DOC_{auto}$$ (3)

To reduce the degrees of freedom, pre-existing relationships between A_254_ and DOC_import_ (a = 4.2 mg^-1^ L) were used [42], and between Fe_coll_ and DOC_Total_ (b = 10; mg^-1^ L) based on a previous study identifying the contribution of Fe^+3^ to colour at A_254_ [11]. Thus, the only variables to be fitted for A_254_ were the slope of DOC_auto_ (c), and the slope of the Freshness Index (β: α) (d). For A_420_ a, b and c were optimized while d was taken from the A_254_ model. Equations were fit by minimizing the sum of squared errors between observed (A_λ_-Observed) and modeled absorbance (A_λ_-Modelled) (Eq. 4) at 254 nm and at 420 nm.

The above color model (Eqs. 1 to 3) was used to predict absorption at two wavelengths (A_254_ and A_420_) at 23 open water sites across Mälaren. Derived parameters (Table S4 in File S1) were then tested on eight Mälaren stream inflows where data on filtered iron were available.

### Evaluation of long-term lake color trends

Average annual A_420_ was calculated for 69 Swedish lakes (in the following designated as “trend lakes”) sampled four times/year between 1990 and 2010. These lakes are distributed across Sweden, and their WRT range from 2 months up to 5.0 years [43]. Trend significance was assessed using the Sen’s slope test [44]. Trend lakes (n=69) were separated into two groups depending on whether pH was above (n=31) or below (n=38) 6.5. We used the a value from equation 3 close to the highest observed change in A_420_ (0.5 yr^-1^) for the trend lakes as a theoretical starting point to predict how DOC_Sum_, DOC_Input_ and Fe_coll_ would change with increasing WRT, using decay constants we established from Mälaren.

## Results

### Historical trends in Mälaren

Both the western highly colored, short WRT and eastern, less colored, longer WRT basins of Mälaren have become browner over the last 50 years. The five year average of filtered absorbance (A_420_) in the short WRT western sampling points doubled within 40 years from 1.6 in 1965 to 3.3 in 2005 (0.046 yr^-1^). Only a fraction of this color increase was measured in the eastern parts of the lake with a longer WRT (corresponding to sampling points within basins C-E and the lake outlet) where A_420_ increased from 0.95 to 1.2 (0.014 yr^-1^).

### Spatial trends across Mälaren

**Figure 3:**
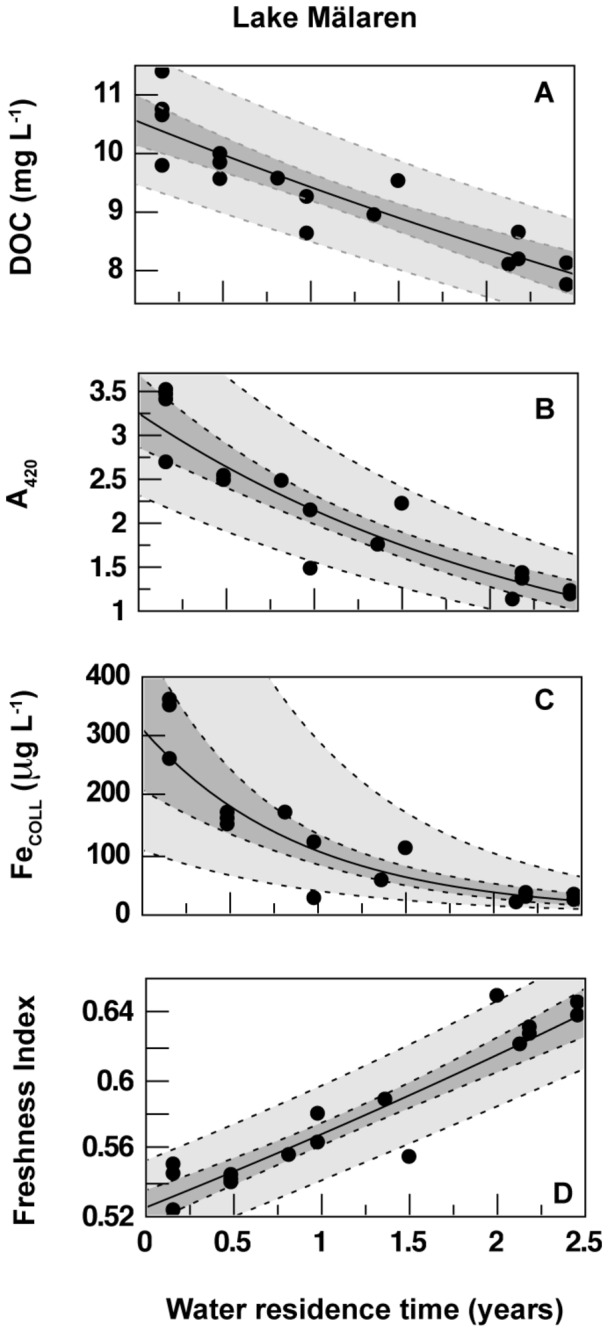
Changes to the concentration of (A) dissolved organic carbon (DOC), (B) absorbance at 420 nm (A_420_), (C) colloidal Fe (Fe_COLL_), and (D) the freshness index with increasing water residence time across Mälaren.

A_420_, A_254_, DOC, TOC and Fe_coll_ decrease when moving from young waters in the western basins A and B, or from the northern basin D to older water in the outlet basin F (Table 1). DOC concentration decreased by 2.9 mg L^-1^ from 10.5 to 7.7 mg L^-1^ between basins A and F (Figure 3a) over the 2.8 years average age of water at the lake outlet. This represents an annual loss rate of 1.0 mg L^-1^ y^-1^ corresponding to a net half-life of 6.1 years (Table 2). In contrast, the decline in A_420_ from 3.26 to 1.01 amounts to an annual loss rate of 0.8, and a much shorter half-life of 1.7 years (Table 2). Thus, despite a loss of DOC of only 26%, we found a 66% and 43% loss in color based on A_420_ and A_254_, respectively. However, the loss in Fe_coll_ was the greatest (95%), from 312 to 15 µg L^-1^ over 2.8 years, with a half-life of only 0.6 years (Table 2, Figure 3c). Thus much of the Fe_coll_ (50%) and A_420_ (30%) was lost between basins A and B with a combined WRT of only 0.67 years (Table 1). Most of the remaining Fe_coll_ was subsequently lost in basin C (Table 1).

**Table 1:** Key chemical and optical characteristics of each of the basins comprising Mälaren including average water retention time (WRT), dissolved organic carbon (DOC), organically-associated iron (Fe_coll_) in each basin, absorbance at 420 and 254 nm (A_420_ and A_254_), the freshness index (β: μ) derived from fluorescence analysis, total organic carbon (TOC), total phosphorous (TP), Chlorophyll and Total Silica (Si_Total_) from the August 2012 sampling campaign.

**Lake Basin**	**A**	**B**	**C**	**E**	**F**	**D**
WRT [years]	0.07	0.6	1.8	0.4	2.8	1.2
	→	→	→	→	Outlet	←
DOC [mg L^-1^]	10.6	9.7	8.6	8.0	7.8	12.7
Fe_coll_ [μg L^-1^]	340	165	55 (34)*	17	58 (21)*	31
DOC:Fe [mg mg^-1^]	31	59	156	470	135	409
A_420_	2.93	2.09	1.35	1.03	1.0	2.16
A_254_	41.4	34.6	26.2	23.3	22.4	42.2
β:α	0.53	0.54	0.60	0.65	0.62	0.59
TOC [mg L^-1^]	11.0	10.6	10.5	10.2	9.2	13.7
P_Total_ [μg L^-1^]	32	38	26	26	18	31
Chlorophyll [μg L^-1^]	41	25	14 (11)*	10	8	13 (9)*
Si_Total_ [mg L^-1^]	0.9	0.8	0.3	0.3	0.1	2.5

The location of lake basins are shown in Figure 2, and arrows indicate the direction of flow between the basins.

* Average and median value of the pooled basin data deviated more than 20% which is why the median value is also given in parenthesis as comparison.

**Table 2:** Description of the exponential change (y = *b* × e^k × age^) in whole dissolved organic carbon (DOC_Sum_) in mg L^-1^, colloidal associated iron (Fe_coll_) in μg L^-1^, absorbance at wavelengths (λ) 420 and 254 nm (A_λ_) (unitless but over path length of 1 m), specific absorbance (SUVA_λ_) in L mg C^-1^ m^-1^, DOC estimated to be from internal microbial (DOC_FI_auto_) and external terrestrial (DOC_FI_import_) sources in mg L^-1^ based on the fluorescence index (FI), the humification index (HIX) and the freshness index (β: α), across the main lake basins (passing from A to F, excluding basin D), including the adjusted *R* ^*2*^, the significance level, (*** *p* < 0.0001, ** *p* < 0.001, * *p* < 0.05), number of sites across the lake (*n*), the amount of a variable at a water residence time of 0 (t_0_), and 2.8 (t_2.8_) years, the net change within the lake (Δ in lake), and the half-life (t_1/2_) (full details of fitting parameters *b* and *k* provided in Table S3 in File S1, as well as results of molecular weight size classes).

	***b***	***k***	**adj. *R*^*2*^**	**sig.-level**	***n***	**t_0_**	**t_2.8_**	**Δ in lake**	**t_1/2_**
DOC_Sum_	10.5	0.114	0.82	***	17	10.5	7.7	-2.9	-6.1
Fe_coll_	312	-1.09	0.80	***	17	312	15	297	-0.6
A_420_	3.3	0.418	0.86	***	17	3.26	1.01	-2.25	-1.7
A_254_	0.4	0.255	0.89	***	17	0.41	0.20	-0.21	-2.7
SUVA_420_	0.3	0.307	0.86	***	17	0.31	0.13	-0.18	-2.3
SUVA_254_	4.1	0.255	0.89	***	17	4.11	2.01	-2.10	-2.7
DOC_FI_auto_	2.5	0.067	0.67	***	16	2.49	3.01	0.51	10.4
DOC_FI_import_	8.0	0.182	0.89	***	17	7.95	4.78	-3.17	-3.8
FI	1.3	0.021	0.82	***	17	1.35	1.43	0.08	
HIX	0.9	0.009	0.42	**	17	0.89	0.87	-0.02	
β:α	0.5	0.079	0.90	***	17	0.52	0.65	0.13	
%Very Large	1.0	0.788	0.61	**	17	1	0	-1	
% Large	24.4	0.139	0.75	***	17	24	17	-8	
%Medium	74.5	0.062	0.83	***	17	75	89	14	
%Small	90.7	0.240	0.06	no fit	17	−	−	−	

### Changes in spectral characteristics of DOM

Observed changes in spectral characteristics (A_λ_, SUVA_λ_, FI, α: β) with increasing WRT were more substantial across the lake (Basins A to F) than expected based on the relatively small loss in DOC (Table 2 and Figure 3). The SUVA_254_ decreased from 4.4 to 2.9 L mg C^-1^ m^-1^, representing a drop in mean aromaticity from 30% to 17%. The FI increased from 1.36 in the western basin, to 1.43 in the eastern basin. Based on changes in the freshness index and equation 2, the proportion of internally produced DOC (DOC_FI_auto_) ranged from 0.28 in the western basin A where the water is youngest to 0.34 in eastern basins. The estimated loss of terrestrial DOC_FI_imports_ equals 4.5 mg L^-1^, which is greater than the net measured loss of DOC (2.8 mg L^-1^). Assuming changes in FI reflect changes in source of DOC, we estimated that DOC_FI_auto_ increased with WRT (0.51 mg DOC L^-1^), and would double within the lake every 10 years, while DOC_import_ was lost (-3.17 mg DOC L^-1^) with increasing WRT, and had a half-life of 3.8 years (Table 2). Both estimates are lake specific and the 10 year doubling time of DOC_FI_auto_ is most probably limited by the supply of nutrients and DOC_import_.

An increase in freshness index from 0.52 to 0.65 with increasing WRT (Figure 3d, Table 2) suggests increased input of internally produced DOC. Very large MW material was found primarily in basins A and B, and was reduced to trace amounts by basin C. Large MW material shifted from 24% to 17% across the lake while the medium MW material shifted from 75% of DOC in basins A and B to 89% in the eastern basins (Table 2). We found no significant change in the small MW size fraction (4% of DOC, Table 2). These results are consistent with the preferential loss of high MW fractions of DOC_import_. We found that the proportion of Peak T fluorescence was positively related to the small DOC size fraction (R^2^ = 0.28, p < 0.05, Figure S2).

The elemental composition of DOM also changed, as shown by generally declining DOC: DON, with basins A, B, C, E and F having DOC: DON of 21, 23, 18, 20 and 15, respectively.

### Statistical analysis of drivers and changes between basins

Linear and multiple linear regressions were used to identify relationships between DOC, A_254_, A_420_, Fe_coll_, and WRT (Table 3 and Table S5 in File S1). A_420_ and DOC were related (M1: R^2^ = 0.58). The best model for A_420_ explained 98% of the variation when using DOC and Fe_coll_ (M2, Table 3). The offset for this equation (M2 with A_420_ of -1.04) suggested the presence of significant amounts of uncolored DOC in Mälaren. Adding WRT as an additional factor did not increase R^2^ but essentially eliminated the offset for A_420_ (M3, Table 3). The contributions of DOC and Fe_coll_ to A_420_ were always positive. When WRT alone was used to predict A_420_, 71% of the variability was explained (M4), and the regression became even stronger when WRT along with Fe_coll_ were used to predict A_420_ (M5, 87%). All A_254_ models corroborated the trends observed for A_420_. Even when using literature values for the coefficients for Fe_coll_ and DOC respectively (M11 and M12) negative offsets were obtained, again suggesting the presence of uncolored DOC.

**Table 3:** Results of selected model (M) parameters and goodness of fits for single and multiple linear regressions predicting absorbance (A_420_ or A_254_) in Mälaren using dissolved organic carbon [mg L^-1^] organically associated dissolved iron [mg L^-1^] and lake water residence time (WRT/1000) for the 23 lake sampling points.

**Model No.**	**Predicted**	**Intercept ± SE**	**DOC**	**Fe_coll_**	**WRT**	**R^2^**	**RMSE**
M1	A_420_	-1.18 ± 0.6	0.316 ± 0.06	excl.	excl.	0.58	0.5
M2	A_420_	-1.04 ± 0.12	0.258 ± 0.01	3.78 ± 0.2	excl.	0.98	0.1
M3	A_420_	-0.049 ± 0.03	0.222 ± 0.03	3.29 ± 0.3	-3.39 ± 2	0.98	0.1
M4	A_420_	2.56 ± 0.01	N.S.	excl.	-16.9 ± 2	0.71	0.3
M5	A_420_	2.07 ± 0.02	excl.	2.7 ± 0.7	-13.6 ± 3	0.87	0.3
M6	A_254_	-10.5 ± 3.99	4.41 ± 0.4	excl.	excl.	0.85	3.2
M7	A_254_	-9.3 ± 1.3	3.99 ± 0.1	25.6 ± 0.1	excl.	0.99	1.1
M8	A_254_	-2.0 ± 2.6	3.54 ± 0.2	18.5 ± 2.0	-1.84 ± 0.5	0.99	0.7
M9	A_254_	12.8 ± 5.10	2.58 ± 0.5	excl.	-4.87 ± 0.8	0.95	1.9
M10	A_254_	41.7 ± 1.10	excl.	N.S.	-8.47 ± 0.8	0.85	3.1
M11	A_254_	-10.6 ± 2.7	4.32 ± 0.27	10^#^	excl.	0.93	2.1
M12	A_254_	-11.2 ± 3.3	4.2^&^	24.7 ± 1.9	excl.	0.89	1.1

The standard error (SE) of the intercept is given, along with the coefficient of variation (R^2^), and the root mean squared error of the regression (RMSE).

N.S. not significant (p > 0.05). excl. = this variable was excluded from that model exercise. ^#^ taken from Weishaar et al. (2003). ^&^ taken from Laudon et al. (2004).

### Estimating the relative contribution of Fe_coll_ and DOC to absorbance

Model 2 (M2-A_420_) from Table 3 was used to estimate the relative contributions of Fe_coll_ and DOC to A_420_ in each basin. The relative contribution of Fe_coll_ to A_420_ decreased from 32% in basin A, to a minimum of 3% in basin E (Table 4). The relative contribution of DOC to A_420_ increased from 68% to 90% between basins A and F (Table 4). The removal of Fe_coll_ from basin A to C explained more of the change in A_420_ than the net loss in DOC in between basins A to C. The remaining changes in A_420_ from basin C to F are caused almost exclusively by decreasing DOC concentrations.

**Table 4:** Comparison of the percent contribution of organically associated iron (Fe_coll_) and dissolved organic carbon (DOC) to the color of water (A_420_) in Mälaren basins during the august 2010 sampling, flow-weighted mean A_420_ from the eight sampled stream inflows, and average A_420_ from each lake basin.

**Lake Basin**	**A**	**B**	**C**	**E**	**F**	**D**
	→	→	→	→	Lake Outlet	←
Mean A_420_ from eight streams	2.87					
Mean A_420_ in Lake Basins	2.93	2.09	1.35	1.03	1.00	2.16
Mean model A_420_ in Lake Basins	2.98	2.08	1.39	1.09	1.19	2.35
Contribution of Fe_coll_ to model A_420_ (%)^&^	32	20	9	3	10	4
Contribution of DOC to model A_420_ (%)^#^	68	80	91	97	90	96

Bold arrows show the direction of water flow from basins to the lake outlet.

^&^ Calculated contribution to M2 by Fe_coll_ only after accounting for the offset in M2. Exemplified for basin A this value (%) is (100*Fe_coll_/1000* 3.78)/(2.98-1.04) = 32. ^#^ calculated contribution to M2 by DOC only after accounting for the offset in M2. Small deviations between the sum of the contributions (M2-A_420_ (Fe_coll_) + M2-A_420_ (DOC)) reflect the remaining error in prediction of M2.

**Figure 4:**
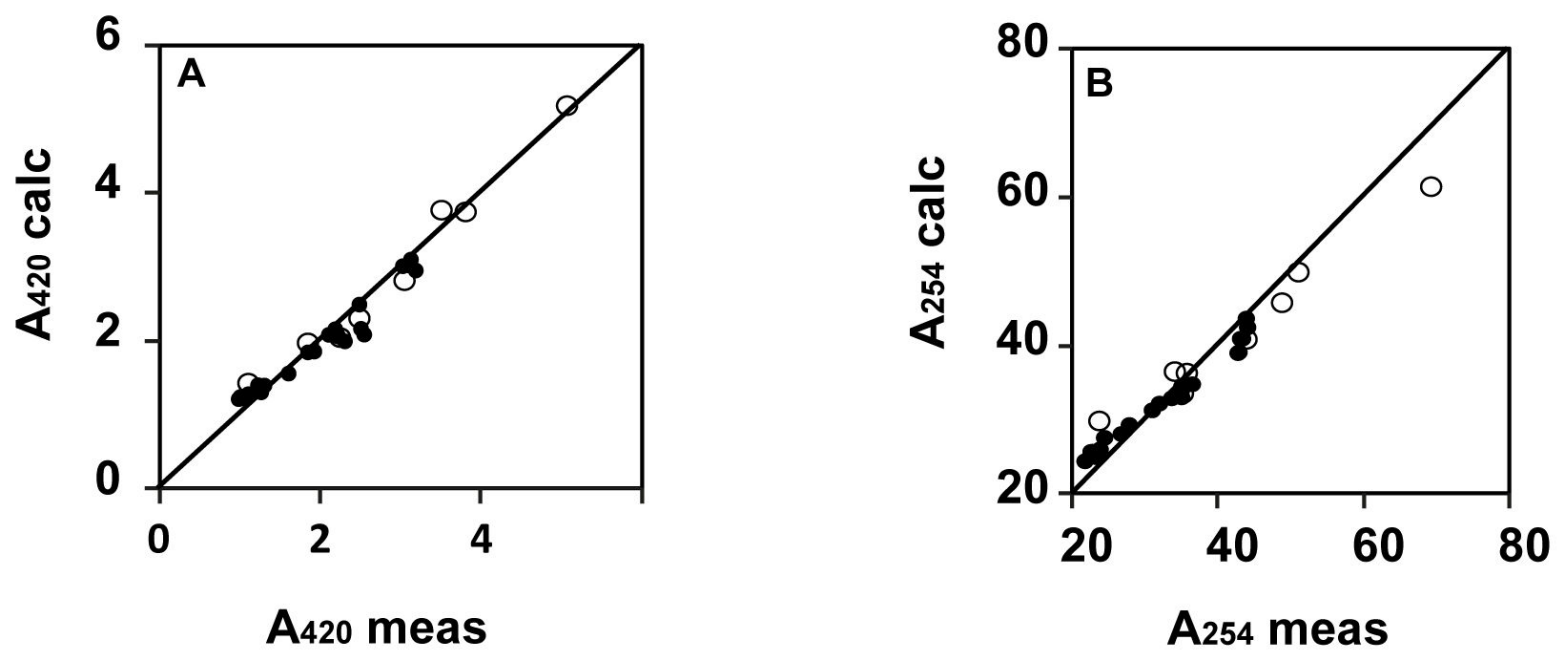
Comparison of observed (meas.) and modeled (calc.) absorbance for the 22 lake sites (•) in Mälaren at wavelengths of 254 nm (A_254_ calc and A_254_ meas) (A) and at wavelength 420 nm (A_420_ calc and A_420_ meas) (B) for the same 22 lake sites. Modeled absorbances were established using Eqs. 1 to 3. In both graphs we used the eight stream inflows (ο) to Mälaren as validation samples using parameter values defined from the lake sites (Table S4 in File S1).

Both A_254_ and A_420_ in Mälaren were predicted from DOC, β:α and SUVA (Figure 4, Table S4 in File S1). The model was corroborated using data from the eight sampled rivers draining into Mälaren (Figure 4a and b)

### Comparison of patterns observed in Mälaren to patterns observed across the 69 Swedish trend lakes

**Figure 5:**
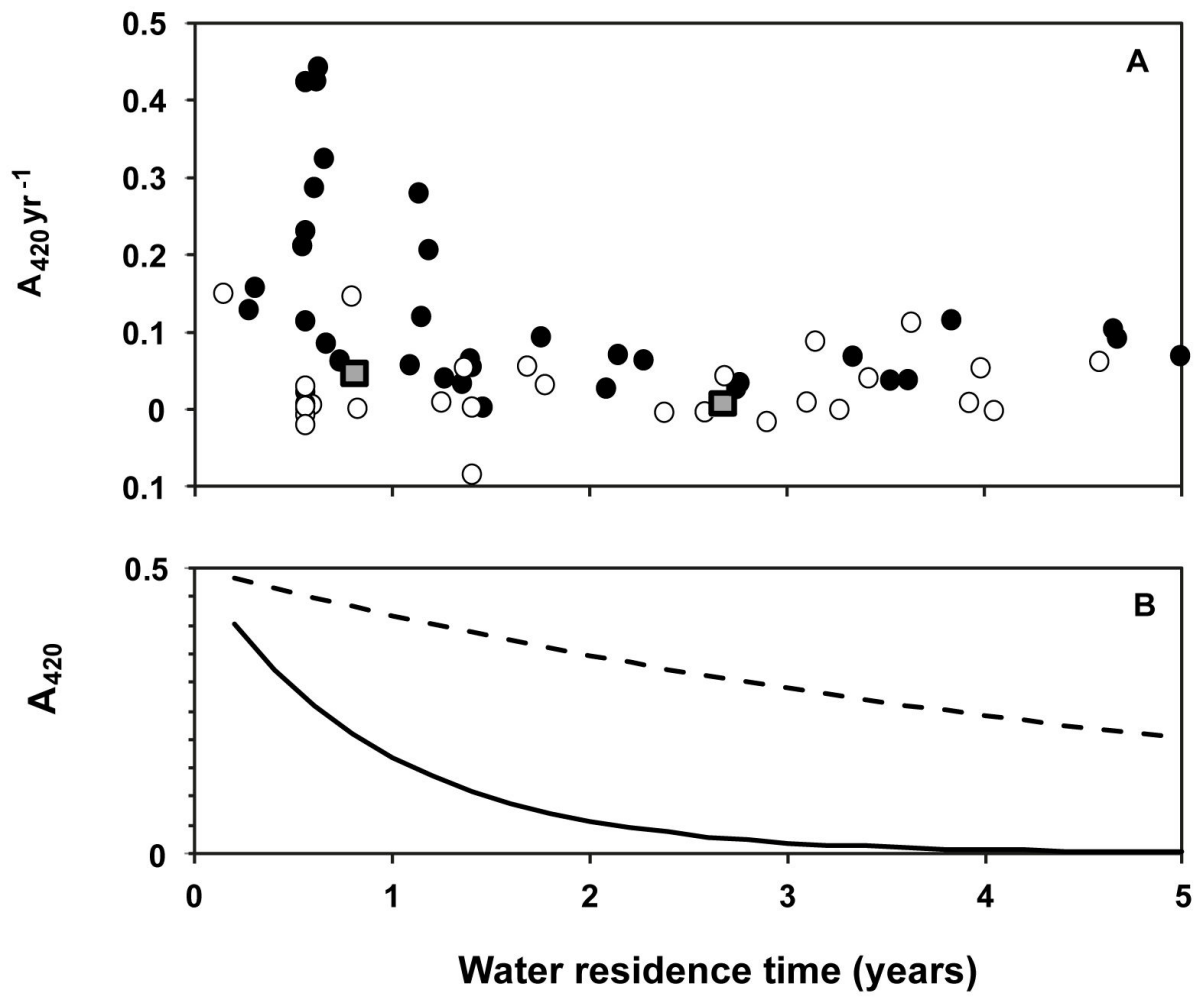
Change in absorbance at 420 nm (Δ A_420_) per year using the Sen’s slope during the period 1990-2010 for 69 trend lakes across Sweden, against water residence time (WRT). (A) Lakes are indicated as those with median lake water pH > 6.5 (white circles ο) and below 6.5 (dark circles •). The two grey squares represent the observed rates of change for the Western (to the left) and Eastern Basins of Mälaren (to the right); (B) and plots of the calculated change in A_420_ caused by either DOC_input_ (hyphenated curve) or Fe_coll_ (bold curve) when starting from a hypothetical value of change in A_420_ of 0.5 yr^-1^ at a WRT of 0 years and using the decay constants for DOC_input_ and Fe_coll_ from table 4 that were derived for Mälaren.

To evaluate whether controls on color (A_420_) in Mälaren were applicable at a wider scale, we included an evaluation of temporal trends from 69 long-term Swedish monitoring trend lakes over 20 years (Figure 5a). A principal component analysis of the drivers of trends in color and TOC in these 69 trend lakes indicates that high pH is correlated with lower color and lower iron content and that high WRT implies low iron, lower color and less iron per DOC (Figure S3). Of the 69 trend lakes, only four had negative change in A_420_ and those with the greatest change in A_420_ were found to i) have a shorter WRT (< 1.5 years), and ii) most were acidic (pH < 6.5) (Figure 5a). We used change in A_420_ of 0.5 yr^-1^ as a theoretical starting point to evaluate how DOC_Sum_, DOC_Input_ and Fe_coll_ changes with WRT, using decay constants from Mälaren (Figure 5b). The decline is steepest in Fe_coll_ and follows the same pattern with WRT observed for changes to A_420_ in acidic (pH < 6.5) trend lakes. The hypothetical change in DOC_Sum_ (Figure 5b; based on a starting A_420_ of 0.5 yr^-1^) and DOC_Input_ decreases with WRT, but not as steeply as Fe_coll_.

## Discussion

Browner waters have been observed across the northern hemisphere [1]. This phenomenon has been ascribed to large increases in DOC [45] [2] and increased iron concentrations, amongst other factors [3]). This study demonstrates that lakes with and without browning trends can occur in the same geographic region. The continuum of WRT across Mälaren (Figure 2), along with the dominant source of terrestrial DOC in the western basin (Table S1 in File S1), allowed us to substitute space for time, and hence assess changes in spectral characteristics of DOC with increasing WRT. This study demonstrates that longer residence times in the eastern basins of Mälaren counteract the increasing inputs of color and DOC observed over the past 40 years. While color has risen by more than 50% in the western part of Mälaren, the eastern lake outlet has experienced a much smaller change (Figure 1) and [46].

In Mälaren, concentrations of colloidal iron decreased much faster than color and DOC when passing from the western to the eastern basin (Table 1 and Figure 3c). The highly colored water entering Mälaren in the western part (Tables S1 and S2 in File S1) does not make it through the lake. Around 60% of the total color loss (loss of A_420_) across Mälaren is occurring simultaneously with the removal of colloidal iron (Table 4). The close relationship between dissolved iron and iron from filtered water samples passing the ion-exchange column (Figure S1) make us believe that the vast majority of all iron in Mälaren is strongly associated to organic matter either as mononuclear iron bound to organic matter or in form of small colloids associated to organic matter. This interpretation is consistent with experimental studies [3,14,47]. Lyven et al. [14] demonstrated the existence of two separate iron phases, one inorganic and one organic in surface water. Jansen et al. [12] reported that iron binds strongly to organic matter and that only a few percent (< 5%) of iron may actually occur as truly dissolved inorganic species.

Our color model suggest that ongoing selective removal of colloidal iron in the western basins may explain much of the absence of changing color in the eastern part of Mälaren (Figures 1, 3b and Table 4). Based on model calculations (Table 1, 3 and Table S2 in File S1), between 2 and 45% of the color in incoming streams may be due to colloidal iron. In Mälaren up to 32% of the color may be assigned to this type of colloidal iron (Table 4). Experiments have shown that iron added to water samples imparts known spectrometric properties such as A_254_ or A_420_ [11,48,10]. When iron was reduced to ferrous iron, large changes in absorbance over the whole UV and visible spectra were observed [49]. Empirical observations of correlations of total iron, DOC and color in both stream [3] and lake waters [9] are consistent with our findings in Mälaren. The color model we used to predict A_254_ (Figure 4b) was based on existing literature [11], and the slope factor used for iron (Table S4 in File S1) in the A_420_ model (Figure 4a) was within 10% of that derived by [3].

### Water age and changes in optical properties of Mälaren waters

The modeled water age depends on a several factors including uncertainty in bathymetry, measured and modeled runoff, wind speed and minor differences in annual rainfall between 2007, 2008 and 2009 [33]. This will affect the calculated changes of optical properties over time for water bodies of intermediate age (1<years<2) but not the very young or very old water. However, general trends and the span of age of water across Mälaren are valid nevertheless.

In our model, lake water optical properties across the lake change as a result of change of abundance of DOC endmembers and colloidal iron. The average optical properties of the two DOC endmembers derived here do not take into account the expected photochemical transformation of those DOC endmembers over time. Despite this simplification our analysis of optical parameters is able to reproduce the changes observed in organic matter character during the 2.8 year lake passage. Results of a simple two-box mixing model using end-member sources (derived from the FI) suggested a loss of half the terrestrial DOM in only 3.8 years, while in contrast autochthonous DOM would be expected to double every 10.4 years. By sampling Mälaren in late summer, we likely captured DOC with maximum processing from photo-decomposition, microbial decomposition, and primary production of new DOC. A larger proportion of protein-like fluorescence (Peak T) relative to humic-like (Peaks A and C) fluorescence with increased WRT supports the concept of a shift in DOC quality to more microbial and algal derived material. Additionally, decreasing DOC: DON with increasing WRT implies a greater proportion of N-containing compounds, which may contribute to the protein-like fluorescence of Peak T. This transformation would be expected as N-poor terrestrial DOM is gradually metabolized and replaced by N-rich autochthonous DOM [50,51]. A strong relationship between Peak T and low MW DOC also suggests that the DON is possibly small molecular weight amino acids and peptides, rather than large macromolecular protein structures. Since autochthonous DOC tends to be less colored than allochthonous material, internally produced DOC can effectively dilute the overall color [52,53]. This is consistent with our observation that the very high MW fraction disappears rapidly within the first lake basin, and the high MW fraction disappears across the lake. The rapidly lost Fe-associated DOC may be of very large molecular weight. Studies of DOC size distribution based on ultrafiltration have shown that the relative importance of high MW DOC decreases with decreasing total DOC concentration across lakes [54]. High MW DOC (>10kD) may be more available than lower-MW DOC as a substrate for bacterial growth, suggesting this fraction is preferentially removed during degradation [54]. Likewise [55], suggest a size-reactivity continuum of DOC, with the larger components being more easily degraded than smaller ones.

Our two estimates of internal primary production of DOC suggest significant formation of autochthonous DOC with increasing WRT. The combination of fluorescence and absorbance may be used to estimate amounts of internally produced DOC. Using our second absorbance model (Table S4 in File S1) we may assign changes in DOC sources to quantitative changes in water color. According to this model, lake color increases with increasing input of allochthonous and iron associated DOC and decreases when terrestrial DOC is replaced by autochthonous DOC (Table 1, 4 and Table S4 in File S1). From this model we conclude that A_420_ is much more strongly affected than A_254_ by the presence of Fe_coll_. While SUVA is more conservative with respect to iron, iron removal strongly affects A_420_ and variations in A_420_ in water bodies with short retention times such as streams are driven by temporal variations in terrestrial iron inputs. A_254_ on the other hand is mainly driven by the relative contribution of DOC_input_ and DOC_lake_ (cf. Table S4 in File S1) and thus mainly controlled by shifts in DOC pools that occur during slow lake DOC processing [55].

### Implications for drinking water treatment plants

Understanding the mechanisms of browning in Mälaren is particularly important since this lake provides drinking water to 1.5 million people in the Stockholm area. Approximately half of the source water DOC (4 to 5 mg/L) is resistant to removal during water treatment, which typically includes a flocculation step. High SUVA is known to facilitate DOC removal [56]. Accordingly, it is possible that water treatment may be able to remove virtually all allochthonous DOC with high SUVA, but not the DOC produced in the lake that has low SUVA. During wet periods SUVA is high when the fraction of allochthonous DOC is high and it decreases during low flow periods where autochthonous DOC is higher. The positioning of the drinking water plants downstream of the five lake basins leads to lower overall DOC but a larger contribution of autochthonous DOC with lower SUVA reduces flocculation efficiency.

### Implications for future and past changes of lake water chemistry in boreal lakes

Time series of 69 lakes distributed across Sweden reveals striking differences in browning trends (Figure 5). Lakes with WRT >1.5 years generally show much lower rates of browning. This is supported by observations from the UK Acid Waters Monitoring network where lakes with WRT<3 months have shown significant increases in DOC [57]. Similar relationships between browning trends and WRT have been observed in Finland [45] and the northeast United States of America [4]. For lakes with WRT times below one year, pH seems to be an additional controlling factor with acidic lakes (pH < 6.5) generally showing up to 5 times higher trends in browning than more neutral to alkaline lakes (pH > 6.5) (Figure 5a and Figure S3). This may be due to the tendency for iron to form particulates at higher pH [34,14,58]. Thus, with increasing pH dissolved iron concentrations decrease leading to a concurrent decrease in filtered absorbance (A_254_ and A_420_). Our studies across lake basins of Mälaren representing a gradient of WRT, as well as 69 lakes with different WRT’s monitored for 20 years suggest that a combination of high pH, high dissolved iron and long WRT strongly counteracts browning (Figure 5a and 5b). As a result, the frequently observed browning of lakes in different geographic regions is expected to be most pronounced in acidic lakes and streams receiving low amounts of colloidal iron and having a short WRT.

## Supporting Information

Figure S1 **Figure A>, Comparison between filtered iron (Fe DISSOLVED) and iron from a filtered water sample passing the ion-exchange column (Fe POSTCOLUMN).**
(TIF)

Figure S2 Relationship between small MW (% of all size fractions) dissolved organic carbon (DOC) and the abundance (%) of PeakT (PeakT/(Peak A + Peak C + Peak M + Peak T) *100) *100) of Peak T across sites in Mälaren (Small MW = 0.57(Peak T) + 0.027, R^2^ = 0.28, n= 22, p < 0.05).
(TIF)

Figure S3 **Principal component analysis of the drivers of change in color and change in TOC of the trend lakes where WRT was available.** As input data average pH, TOC, A420, total iron (Fe), total iron per carbon, (Fe/TOC), annual change in A420, annual change in TOC and WRT.
(TIF)

File S1 **Supplementary information.** Table **S1**, Basin coding and average water retention time (WRT) in each basin. A comparison of a number of measured parameters (A420, TOC, total iron, total silica, total phosphorous and chlorophyll) of the regular lake monitoring program for the six lake basins in Lake Mälaren from the 2010 sampling campaign, mean values for the corresponding data over 3 years between 2007–2009 in parenthesis (upper part) and selected additional parameters of the august 2010 sampling (lower part). Arrows indicate the direction of flow between the basins. Table **S2**, Summary of stream coding, flow contribution, catchment area and average TOC and A420 in order of greatest hydrological contribution and the average flow weighted contribution (STREAM) of the six streams with available data (i.e. excluding S4 and S6) over 12 years (1998-2009), the last 3 years (2007-2009) and the august 2010 sampling for A420 (TOC) during respective period. Table **S3**, Key variables for eight primary streams inflows to Lake Mälaren including organically associated iron (Feorg), dissolved organic carbon (DOC), absorbance at 420 measured (A420-Measured) and modeled (A420-model), and the relative contribution of DOC and Fecoll to A_420_. Table **S4**, Parameter values used to predict A420-Modelled and A254-Modelled based on Equations 1 to 4, using freshness index (β: α), dissolved iron (Fecoll) and proportion of stream (DOCimport) and lake dissolved organic carbon (DOCauto). Table **S5**, Pairwise correlations of drivers used for color models.
(DOCX)
