# Supplementary figures and images for: In-Lake Processes Offset Increased Terrestrial Inputs of Dissolved Organic Carbon and Color to Lakes
Source: PLoS One. 2013 Aug 15;8(8):e70598. doi: 10.1371/journal.pone.0070598 (PMC3744563; doi:10.1371/journal.pone.0070598)

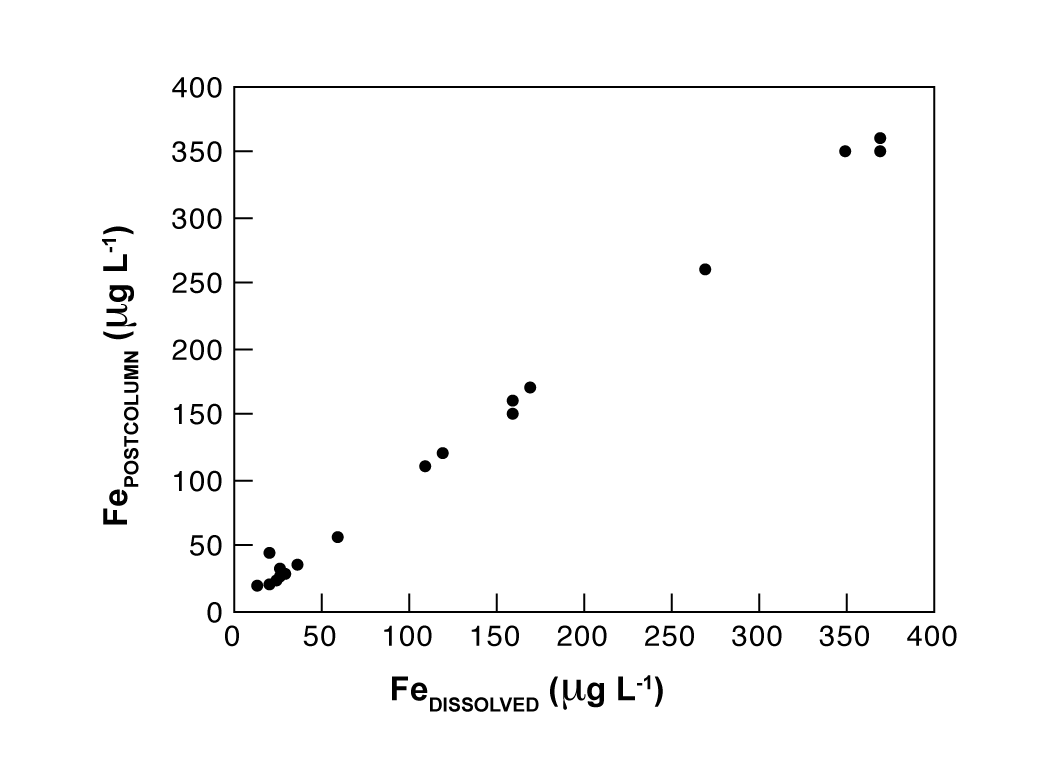

Supplement: Figure S1 — Figure A>, Comparison between filtered iron (Fe DISSOLVED) and iron from a filtered water sample passing the ion-exchange column (Fe POSTCOLUMN). (TIF) [file pone.0070598.s001.tif]

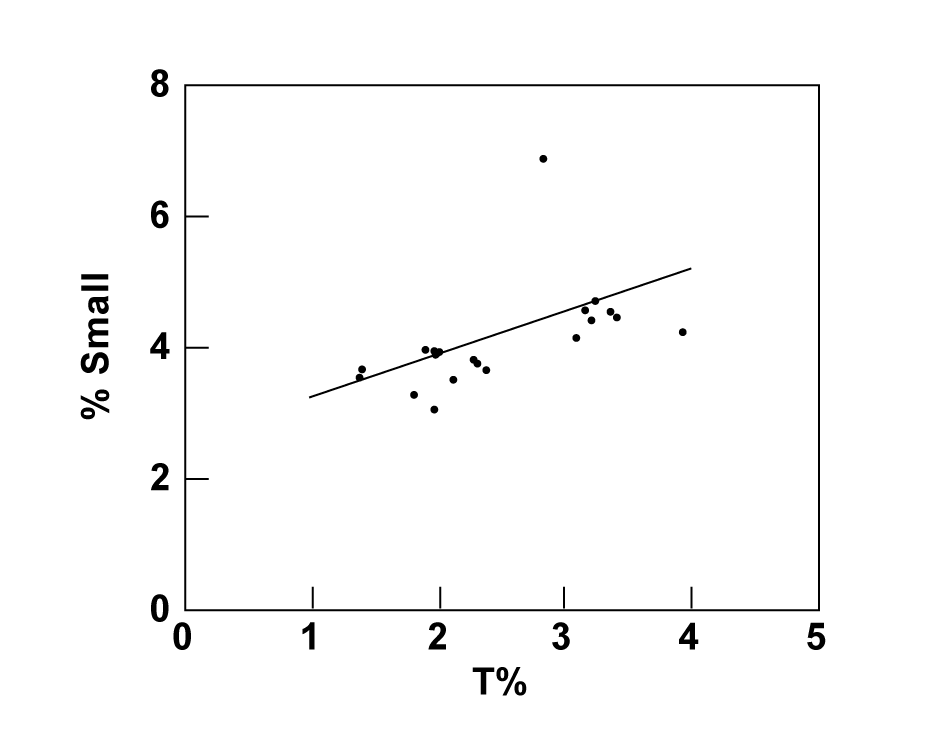

Supplement: Figure S2 — Relationship between small MW (% of all size fractions) dissolved organic carbon (DOC) and the abundance (%) of PeakT (PeakT/(Peak A + Peak C + Peak M + Peak T) *100) *100) of Peak T across sites in Mälaren (Small MW = 0.57(Peak T) + 0.027, R2 = 0.28, n= 22, p < 0.05). (TIF) [file pone.0070598.s002.tif]

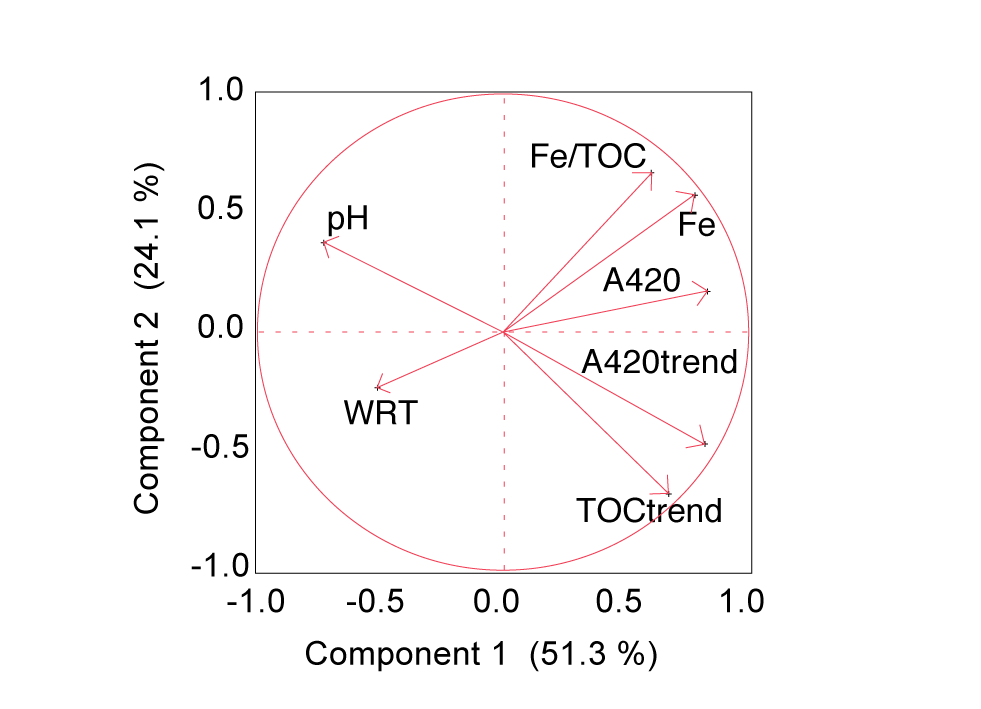

Supplement: Figure S3 — Principal component analysis of the drivers of change in color and change in TOC of the trend lakes where WRT was available. As input data average pH, TOC, A420, total iron (Fe), total iron per carbon, (Fe/TOC), annual change in A420, annual change in TOC and WRT. (TIF) [file pone.0070598.s003.tif]
