# Supplementary material for: In-Lake Processes Offset Increased Terrestrial Inputs of Dissolved Organic Carbon and Color to Lakes
Source: PLoS One. 2013 Aug 15;8(8):e70598. doi: 10.1371/journal.pone.0070598 (PMC3744563; doi:10.1371/journal.pone.0070598)
Supplement: File S1 — Supplementary information. Table S1 , Basin coding and average water retention time (WRT) in each basin. A comparison of a number of measured parameters (A420, TOC, total iron, total silica, total phosphorous and chlorophyll) of the regular lake monitoring program for the six lake basins in Lake Mälaren from the 2010 sampling campaign, mean values for the corresponding data over 3 years between 2007–2009 in parenthesis (upper part) and selected additional parameters of the august 2010 sampling (lower part). Arrows indicate the direction of flow between the basins. Table S2 , Summary of stream coding, flow contribution, catchment area and average TOC and A420 in order of greatest hydrological contribution and the average flow weighted contribution (STREAM) of the six streams with available data (i.e. excluding S4 and S6) over 12 years (1998-2009), the last 3 years (2007-2009) and the august 2010 sampling for A420 (TOC) during respective period. Table S3 , Key variables for eight primary streams inflows to Lake Mälaren including organically associated iron (Feorg), dissolved organic carbon (DOC), absorbance at 420 measured (A420-Measured) and modeled (A420-model), and the relative contribution of DOC and Fecoll to A420. Table S4 , Parameter values used to predict A420-Modelled and A254-Modelled based on Equations 1 to 4, using freshness index (β: α), dissolved iron (Fecoll) and proportion of stream (DOCimport) and lake dissolved organic carbon (DOCauto). Table S5 , Pairwise correlations of drivers used for color models. (DOCX) [file pone.0070598.s004.docx]

**Supplementary Information for**

# In-lake processes offset increased terrestrial inputs of dissolved organic carbon and color to lakes.

Stephan J. Köhler^1,*^, Dolly Kothawala^2^, Martyn N. Futter^1^, Olof Liungman^3^ and Lars Tranvik^2^

**Supplementary Information**

**Mass Balance of Lake Mälaren Streams.** Using the long-term data, annual mass balances were calculated for the six major stream inflows, accounting for 68% of the total incoming water to the lake, and the outflow based on discharge normalized values using the macro FLOWNORM developed by Grimvall (2004). Yearly fluxes for DOC and A_420_ were calculated from routine monthly stream sampling using either measured or modeled flow supplied by the Swedish meteorological service (SMHI) at the six stations. For some years missing flow data for some sites were estimated from correlations with neighboring sites. In the mass balance, the remaining 30% of the ungauged catchment area was set to behave like the 70% with existing data. Water balance calculations from Lake Mälaren indicate that the net water flux through Mälaren is mainly driven by stream water input (([Wallin *et al.*, 2000](#_ENREF_9))) and only 8% of the water comes from diffuse inputs.

Land cover in the Mälaren catchment (22,603 km^2^) comprises forest (48%), wetland (2%), arable land and meadows (36%), lakes (10%) and urban areas (4%) ([Hagner *et al.*, 2005](#_ENREF_3))

### Suspended Solids. Suspended solids were determined by filtering 1L of lake water using pre-washed and pre-weighted filters and gravimetrically measuring the mass of solids retained on filters after drying at 60 °C for 30 mins.

**Total inorganic carbon.** Immediately upon return to the lab from sampling, unfiltered water samples were added headspace free to 40 mL glass sampling vials with Teflon coated septa. Dissolved inorganic carbon (DIC) samples were stored at 4°C and analyzed within four days using a Sievers 900 portable TOC and TIC analyzer (General Electric).

**Microbial Biomass.** Microbial biomass was measured using a CyFlow space flow cytometer (PARTEC) by preserving 900 μL of unfiltered lake water in 100 μL of sterile filtered (0.2 μm) formaldyhyde (37%).

**Dissolved and organically bound iron, aluminium and manganese**

Total and dissolved iron (Fe), aluminium (Al) and manganese (Mn) were determined from aliquots of the 1.2 μm filtered water samples. Organically bound iron (Fe_org_) and aluminum (Al_org_) were estimated by passing a filtered water sample (250mL) through a proton saturated ion-exchange column (Dowex 50W-X8) following the method of ([Hruska *et al.*, 1996](#_ENREF_4)). The strong cation-exchanger will bind all weakly bound metals or metals occurring as inorganic complexes while metals that are strongly associated with colloidal natural organic matter will move through the column. As the weakly bound base cations (Ca, Mg, Na and K) present in the water sample will bind to the exchanger, the released protons will decrease pH in the sample from around neutral conditions to a pH between 3.8 and 4.0 depending on the initial amount of cations present. On the other hand all iron, manganese and aluminium that is measured in the sample that has passed the ion-exchange column is considered to be strongly associated with organic matter. In oxic waters such as those considered here all iron may be assumed to occur as ferric iron (Fe^3+^).

### High Performance Size Exclusion Chromatography. Size separation was based on a Waters YMC200 silica diol column (Waters Associates, Milford, MA). Molecular weight (MW) was calibrated with polystyrene sulfonate standards with MW of 2.69 x 10^6^, (American Polymer standards, Mentor, OH) 1,048 (Polymer standards service, Amherst, MA) and 210 Da (Fluka). The mobile phase was 0.1 M NaCl (Fisher Scientific, Göteborg), 0.002 M K_2_HPO_4_ and 0.002 M KH_2_PO_4_ (Sigma-Aldrich, Sweden) as previously used by ([Chin et al., 1994](#_ENREF_1), [Kothawala et al., 2006](#_ENREF_5)). After testing the relationship between chromatogram area and DOC concentration (R^2^ = 0.96, p < 0.0001), we divided the chromatogram area into four size classes, very large MW (> 4,000 Da), large MW (1,000 to 4,000 Da), medium MW (200 to 1,000 Da), and small MW (200 Da) molecules. The proportion of DOC in each size class was related to the water residence time of lake water to examine how the molecular weight distribution (MWD) of DOC shifted across the lake.

**Flourescence measurement**

EEMs were blank-subtracted using the EEM of pure water (Milli-Q) run on the same day. Manufacturer supplied correction factors were used to correct excitation and emission intensities for instrument-specific biases (([Coble *et al.*, 1993](#_ENREF_2))), and inner filter effects were corrected using the absorbance spectrum (([Lakowicz, 2006](#_ENREF_6))). Fluorescence intensity was adjusted to Raman units, by dividing by the Raman area of pure water, collected daily, and integrated at λ_ex_ of 350 nm over λ_ex_ of 380 to 420 nm (([Lawaetz & Stedmon, 2009](#_ENREF_8))). All samples were run in signal (S) mode and were corrected for lamp excitation reference intensity (R) by scaling to the recorded lamp intensity.

**Comparison of August 2010 sampling campaign to three year and 12 year averages.**

The August 2012 sampling campaign was compared to A) three year averages prior to sampling i.e. at the time scale of the turnover time of Lake Mälaren (Table 1) and B) to both shorter (3 year) and longer (12 years) fluxes of stream DOC into Lake Mälaren (Table S1). Lake TOC values from the August sampling were within 10-25% of the three year means (2007-2009) (Table 1). A_420_ and Fe_tot_ in both stream and lake water samples were found to differ substantially (Table 1 and Table 1S). This is in accordance with the observation that stream water flowing into Mälaren was darker during the last three year period (A_420_ = 5.4) than before (A_420_ = 4.1) and during (A_420_ = 2.9) our sampling. This causes higher A_420_ to occur at places where high color input occurs i.e. in the western basins (Basins A and B) and basin D while the differences decrease eastwards (Basin C, E and F). Three year (14.8 mg L^-1^) and twelve year stream flow weighted TOC input (12.2 mg L^-1^) compare well to the august sampling flow weighted TOC (13.3 mg L^-1^). Together with the calculated average outgoing TOC in Table S1 of 7.8 this corresponds to a 3.7 mg L^-1^ (36%) net removal for TOC, slightly higher than what was calculated across the lake from the August 2010 sampling (2.8 mg L^-1^). The generally lower stream TOC and A_420_ (2.9) values (Table S1) as compared to the long-term flow weighted means of A_420_ (4.1) are due to the low flow situation prior to and during sampling. So while significant differences for A_420_ and Fe occurred during the last three years the general trend is in accordance with our August 2010 sampling. These higher A_420_ values within the lake are may be directly related to the higher lake iron concentration in past three years (2007-2009) as will be discussed below.

**Modeling the age of water in Mälaren**

## The model, implemented in the software package MIKE 3 FM, solves the hydrostatic Navier-Stokes equations on an unstructured mesh with a free water surface. The set of model equations is closed using the k-ε turbulence model. In the vertical, a combination of sigma-layers at the surface and z-layers deeper down are used, which in the deepest parts of Mälaren amounts to a maximum of 23 layers. The model includes forcing by the wind, runoff from land, evaporation/precipitation, atmospheric heat exchange, baroclinic and barotropic forces, bottom friction and the Coriolis effect. Inflows to Mälaren are determined by measured and computed runoff. The downstream boundary conditions are given by measured flows and measured water levels. Meteorological observations from a station on the island Adelsö in Mälaren was used to define the exchange of momentum and heat through the water surface. In addition to investigating the general circulation in terms of currents, stratification and net and gross flows between different sub-basins, an age tracer was used to calculate the residence time. To ensure that the initial values of the age tracer are realistic, and because the residence time in some parts of Mälaren exceeds one year, the age tracer was spun up from zero by running repeated one-year simulations of 2007, using the final values from one simulation to initialize the following simulation. Water age did not vary significantly with depth. Note that in a few sub-basins the residence time may exceed three years as in these areas the model shows age concentrations that are above 900 days and still increasing.

**References for Supplementary Methods**

Chin Y-P, Aiken G, O'loughlin E (1994) Molecular weight, polydispersity, and spectroscopic properties of aquatic humic substances. *Environmental Science and Technology,* **28**, 1853-1858.

Coble PG, Schultz CA, Mopper K (1993) Fluorescence contouring analysis of DOC intercalibration experiment samples - A comparison of techniques. *Marine Chemistry,* **41**, 173-178.

Hagner O, Nilsson M, Reese H, Egberth M, Olsson H (2005) Procedure for classification of forests for CORINE land cover in Sweden. New Strategies for European Remote Sensing. pp Page. Rotterdam, Millpress.

Hruska J, Johnson CE, Kram P (1996) Role of organic solutes in the chemistry of acid-impacted bog waters of the western Czech Republic. *Water Resources Research,* **32**, 2841-2851.

Grimvall, A. 2004. FLOWNORM 2.0–A visual basic program for computing riverine loads of substances and extracting anthropogenic signals from time series of load data. Linköping:Department of Mathematics, Linköping University.

Kothawala DN, Evans RD, Dillon PJ (2006) Changes in the Molecular Weight Distribution of Dissolved Organic Carbon Within a Precambrian Shield Stream. *Water Resources Research,* **42**.

Lakowicz JR (2006) *Principles of Fluorescence Spectroscopy,* New York, Springer.

Laudon H, Kohler S, Buffam I (2004) Seasonal TOC export from seven boreal catchments in northern Sweden. *Aquatic Sciences,* **66**, 223-230.

Lawaetz AJ, Stedmon CA (2009) Fluorescence intensity calibration using the Raman scatter peak of water. *Applied Spectroscopy,* **63**, 936-940.

Wallin M, Andersson B, Johnson R, Kvarnäs H, Persson G, Wehenmeyer G, Willen E (2000) Mälaren miljötillstånd och utveckling 1965-98 (in swedish). pp Page, Uppsala, Sweden.

Weishaar JL, Aiken GR, Bergamaschi BA, Fram MS, Fujii R, Mopper K (2003) Evaluation of specific ultraviolet absorbance as an indicator of the chemical composition and reactivity of dissolved organic carbon. *Environmental Science & Technology,* **37**, 4702-4708.

**Supplementary Tables:**

**Table S1: Basin coding and average water retention time (WRT) in each basin. A comparison of a number of measured parameters (A_420_, TOC, total iron, total silica, total phosphorous and chlorophyll) of the regular lake monitoring program for the six lake basins in Lake Mälaren from the 2010 sampling campaign, mean values for the corresponding data over 3 years between 2007-2009 in parenthesis (upper part) and selected additional parameters of the august 2010 sampling (lower part). Arrows indicate the direction of flow between the basins.**

| Basin | A | | B | | C | | E | F | D |
| --- | --- | --- | --- | --- | --- | --- | --- | --- | --- |
| WRT [years] | 0.07 | | 0.6 | | 1.8 | | 0.4 | 2.8 | 1.2 |
|  | 🡺 | | 🡺 | | 🡺 | | 🡺 | Outlet | 🡸 |
| **August sampling (3 year averages (2007-2009))** | | | | | | | | | |
| A_420_ | 2.93 (3.6) | | 2.09 (3.0) | | 1.35 (1.6) | | 1.03 (1.2) | 1.0 (n.d.) | 2.16 (2.8) |
| TOC [mg L^-1^] | 11.0 (11.8) | 10.6 (10.7) | | 10.5 (9.1) | | 10.2 (9.6) | | 9.2 (n.d.) | 13.7 (14.8) |
| Fe_TOTAL_  [μg L^-1^] | 644 (n.d.) | 373 (806) | | 138 (203) | | 111 (n.d.) | | 55 (n.d.) | 79 (502) |
| Si_tot_ [mg L^-1^] | 0.9 (2.7) | 0.8 (3.1) | | 0.3 (1.4) | | 0.3 (1.3) | | 0.1 (n.d.) | 2.5 (5.5) |
| P_tot_  [ppb] | 32 (50) | 38 (47) | | 26 (34) | | 26 (28) | | 18 (n.d.) | 31 (51) |
| Chloro. [ppb] | 41 (21) | 25 (13) | | 14 (13) | | 10 (10) | | 8 (n.d.) | 13 (9) |

**Table S2**: Summary of stream coding, flow contribution, catchment area and average TOC and A_420_ in order of greatest hydrological contribution and the average flow weighted contribution (STREAM) of the six streams with available data (i.e. excluding S4 and S6) over 12 years (1998-2009), the last 3 years (2007-2009) and the august 2010 sampling for A_420_ (TOC) during respective period.

| Name, code and (basin) | Arbogaån  S7-(A) | Kolbäcksån  S5-(A) | Eskilstunaån  S8-(B) | Svartån  S4-(B) | | Hedströmmen  S6-(A) | Fyrisån  S1-(D) | Sagån  S3-(B) | Örsundaån  S2-(D) | Stockholm  (F) |
| --- | --- | --- | --- | --- | --- | --- | --- | --- | --- | --- |
| Q_contr_ [%] | 25 | 17 | 14 | 12 | | 12 | 8 | 4 | 3 | 100 |
| Area [km^2^] | 3802 | 3093 | 4187 | 754 | | 1058 | 1982 | 865 | 727 | 21507 |
|  | **12 year flow weighted averages (1998-2009)** | | | | | | | | | |
| A_420_ | 7.2 ± 1.8 | 2.6 ± 0.4 | 2.1 ± 1.4 | n.d. | | n.d. | 4.3 ± 0.7 | 5.2 ± 1.3 | 5.6 ± 1.1 | 1.0± 0.1 |
| TOC [mg L^-1^] | 11.4 ± 1.1 | 9.4 ± 0.9 | 9.3 ± 0.9 | n.d. | | n.d. | 16.3 ± 2.4 | 14.0 ± 2.5 | 13.3 ± 1.2 | 7.8 ± 0.7 |
| STREAM | 4.1±1.1 (12.2±8.8) | | | | | | | | | |
|  | **3 year flow weighted averages (2007-2009)** | | | | | | | | | |
| Abs_420_ | 7.0 ± 1.9 | 2.9 ±1.0 | 1.2^&^ | n.d. | n.d. | | 4.7 ± 0.7 | 6.2 ± 1 | 6.6 ± 1.2 | 0.92 ± 0.30 |
| TOC [mg L^-1^] | 11.9 ± 1.1 | 10.2 ±1.2 | 10.6^&^ | n.d. | n.d. | | 18.6 ± 3.9 | 15.8 ± 2.8 | 14.0 + 0.9 | 8.4 ± 1.0 |
| STREAM | 5.4±1.1 (14.8±9.3) | | | | | | | | | |
|  | **August 2010 sampling** | | | | | | | | | |
| A_420_ | 3.8 | 2.5 | 1.1 | 5.1 | 3.5 | | 3.0 | 2.2 | 1.8 | 1.1 |
| TOC [mg L^-1^] | 14.3 | 11.7 | 11.4 | 19.0 | 12.0 | | 16.7 | 12.0 | 12.4 | 8.6 |
| Fe_coll_ [μg L^-1^] | 130 | 65 | 13 | 180 | 110 | | 50 | 48 | 38 | n.d. |
| DOC [mg L^-1^] | 12.4 | 9.5 | 9.5 | 16.6 | 10.5 | | 15.1 | 11.0 | 11.1 | n.d. |
| STREAM | 2.9 (13.3) | | | | | | | | | |

^&^ Date from 2008 only and n.d. no data available for the period 2007-2009.

**Table S3:** Key variables for eight primary streams inflows to Lake Mälaren including organically associated iron (Fe_org_), dissolved organic carbon (DOC), absorbance at 420 measured (A_420_-Measured) and modeled (A_420_-model), and the relative contribution of DOC and Fe_coll_ to A_420._.

| **Stream name, code and (basin)** | **Arbogaån**  **S7-(A)** | **Kolbäcksån**  **S5-(A)** | **Eskilstunaån**  **S8-(B)** | **Svartån**  **S4-(B)** | **Hedströmmen**  **S6-(A)** | **Fyrisån**  **S1-(D)** | **Sagån**  **S3-(B)** | **Örsundaån**  **S2-(D)** |
| --- | --- | --- | --- | --- | --- | --- | --- | --- |
| Fe_coll_ [μg L^-1^] | 130 | 65 | 13 | 180 | 110 | 50 | 48 | 38 |
| DOC [mg L^-1^] | 12.4 | 9.5 | 9.5 | 16.6 | 10.5 | 15.1 | 11.0 | 11.1 |
| A_420 (meas)_ | 3.8 | 2.5 | 1.1 | 5.1 | 3.5 | 3.0 | 2.2 | 1.8 |
| A_420 (model)_ | 4.0 | 2.3 | 1.5 | 6.0 | 3.9 | 3.4 | 2.2 | 2.2 |
| % Contribution^#^ of DOC to A_420_ | 63 | 74 | 98 | 61 | 55 | 87 | 87 | 90 |
| % Contribution^&^ of Fe_coll_ to A_420_ | 37 | 26 | 2 | 39 | 45 | 13 | 13 | 10 |

^&^ calculated contribution to M2 by Fe_coll_ only after accounting for the offset in M2. Exemplified for S7-(A) this value (%) is (100*130/1000* 3.78)/(4.0-1.04) = 37.

^#^ calculated contribution to M2 by DOC only after accounting for the offset in M2. Small deviations between the sum of the contributions (M2-A_420_ (Fe_coll_) + M2-A_420_ (DOC)) reflect the remaining error in prediction of M2.

**Table S4**: Parameter values used to predict A_420_-Modelled and A_254_-Modelled based on Equations 1 to 4, using freshness index (β:α), dissolved iron (Fe_coll_) and proportion of stream (DOC_import_) and lake dissolved organic carbon (DOC_auto_).

| **Slope Predicted [unit]** | **a [mg^-1^ L]** | **b [mg^-1^ L]** | **c [mg^-1^ L]** | **d [ ]** |
| --- | --- | --- | --- | --- |
| Parameter | DOC_import_ | Fe_coll_ | DOC_auto_ | β:α |
| A_254_-Modelled | 4.2^*^ | 10** | 0.91 | 0.5 |
| A_420_-Modelled | 0.203 | 3.66 | 0.030 | 0.5 |

* Value derived from ([Laudon *et al.*, 2004](#_ENREF_7)).

** Value derived from ([Weishaar *et al.*, 2003](#_ENREF_10)).

**Table S5 :** Pairwise correlations of drivers used for color models.

| **Variable** | **by Variable** | **Correlation** | **Count** | **Lower 95%** | **Upper 95%** | **Signif Prob** |
| --- | --- | --- | --- | --- | --- | --- |
| DOC [mg L^-1^] | age [years] | -0.7818 | 20 | -0.9096 | -0.5188 | <.0001* |
| Fe_coll_ [μg L^-1^] | age [years] | -0.6110 | 21 | -0.8250 | -0.2435 | 0.0033* |
| Fe_coll_ [μg L^-1^] | DOC [mg L^-1^] | 0.2900 | 22 | -0.1499 | 0.6341 | 0.1905 |
| A254 | age [years] | -0.9227 | 20 | -0.9694 | -0.8116 | <.0001* |
| A254 | DOC [mg L^-1^] | 0.9230 | 23 | 0.8246 | 0.9672 | <.0001* |
| A254 | Fe_coll_ [μg L^-1^] | 0.6105 | 22 | 0.2543 | 0.8208 | 0.0026* |
| A420 | age [years] | -0.8437 | 48 | -0.9098 | -0.7361 | <.0001* |
| A420 | DOC [mg L^-1^] | 0.7631 | 23 | 0.5120 | 0.8941 | <.0001* |
| A420 | Fe_coll_ [μg L^-1^] | 0.7735 | 24 | 0.5380 | 0.8970 | <.0001* |
| A420 | A254 | 0.9469 | 23 | 0.8770 | 0.9776 | <.0001* |
